# How a moderated online discussion forum facilitates support for young people with eating disorders

**DOI:** 10.1111/hex.12439

**Published:** 2016-01-03

**Authors:** Sarah Kendal, Sue Kirk, Rebecca Elvey, Roger Catchpole, Steven Pryjmachuk

**Affiliations:** ^1^ School of Human and Health SciencesUniversity of HuddersfieldHuddersfieldUK; ^2^ School of Nursing Midwifery and Social WorkUniversity of ManchesterManchesterUK; ^3^ Centre for Primary CareUniversity of ManchesterManchesterUK; ^4^ YoungMindsLondonUK

**Keywords:** eating disorders, moderated online discussion, netnography, qualitative, social media, youth

## Abstract

**Introduction:**

Young people with eating disorders are at risk of harm to their social, emotional and physical development and life chances. Although they can be reluctant to seek help, they may access social media for information, advice or support. The relationship between social media and youth well‐being is an emotive subject, but not clearly understood. This qualitative study aimed to explore how young people used a youth‐orientated, moderated, online, eating disorders discussion forum, run by an eating disorders charity.

**Methods:**

We applied a netnographic approach involving downloading and thematically analysing over 400 messages posted August–November 2012.

**Results:**

Data analysis generated five themes: Taking on the role of mentor; the online discussion forum as a safe space; Friendship within the online forum; Flexible help; and Peer support for recovery and relapse prevention. Forum moderation may have influenced the forum culture.

**Discussion:**

Our findings are consistent with literature about youth preferences for mental health self‐care support. A young person's decision to use this discussion forum can be construed as pro‐active self‐care. A moderated online discussion forum can make a positive contribution to support for youth with eating disorders, countering negative media perceptions of online groups.

**Conclusion:**

This study adds to knowledge about how young people access support via social media. Online discussion forums can be safe and acceptable spaces for youth to access help. Further research could provide insights into the impact of forum moderation.

## Introduction

This paper is concerned with online support for young people (10–19 years^1^) with eating disorders. European data suggest a prevalence for eating disorders of up to 0.9% in 14–17 year olds.^2^ This is probably an underestimate^3^, ^4^ but is consistent with data from the World Health Organization.^5^ There is evidence that prevalence is increasing.^6^ Eating disorders can harm emotional, psychological, social and physical well‐being and development.^7^, ^8^, ^9^, ^10^ They are complex conditions, in which self‐worth is judged by weight or body shape. An individual may experience intense fear of fatness linked with a focused pursuit of thinness. They may develop a cycle of overeating followed by induced vomiting or excessive use of laxatives, often accompanied by self‐loathing and extreme anxiety.^1^, ^8^, ^11^

Within the eating disorders community there have been attempts to promote an ideal thinness amongst individuals with eating disorders. This is evident in ‘pro Ana’, ‘pro‐Mia’ and ‘Anamia’ websites,^12^ which equate thinness with self‐control and beauty, often using pictures of celebrities and quasi‐spiritual messages to encourage users to strive for weight loss, no matter the consequences.^13^, ^14^, ^15^, ^16^ Such ideas may be presented as having moral currency, in that the pursuit of thinness is sold as a valid philosophical and moral position.^17^

Developmental theories present adolescence as a phase of growth and exploration, moving towards a sense of identity. According to egosyntonic theory, eating disorders are tightly bound up with identity, thus, they are hard to treat because treatment implies a threat to identity.^18^ A young person's support needs complicate the picture because they vary in response to changing life‐skills, experience, maturity and insight.^18^, ^19^, ^20^ Hence, a complex pattern of treatment resistance is further complicated by developmental factors.^18^, ^21^, ^22^

The personal development and circumstances of young people influence their helpseeking, and limits to privacy, money, transport or freedom of movement can be effective obstacles to accessing services.^23^, ^24^, ^25^, ^26^ Studies from diverse populations (e.g. in Africa,^27^ Australia,^23^ Canada,^28^ Finland,^29^ Japan,^30^ the UK^31^ and the USA^32^) suggest that young people may not disclose emotional health difficulties to others, preferring instead to manage these independently. If their attempts at self‐management are unsuccessful then their help‐seeking decision making is highly dependent on their appraisal of the trustworthiness and effectiveness of a potential confidante. The help‐seeking journey therefore represents many careful judgements between self‐awareness and action. This level of analysis is emphasized by Liang *et al*.^33^ in a three‐phase model of help‐seeking comprising: defining the problem, deciding to seek help and selecting a source of support. The implications for service development are that young people exercise agency in their health behaviour,^25^ and for meaningful engagement with an offer of help, a young person would need to access it on their own terms. Thus there are inherent challenges to supporting an individual experiencing the egosyntonic impact of an eating problem, combined with the developmental and contextual circumstances of youth.

Despite the barriers, young people are strongly motivated to foster their own well‐being^25^, ^34^ so it is important to explore strategies that support this. One possibility is via the Internet, because digital media are part of the social fabric for young people^35^ and could offer acceptable and accessible emotional support. The evidence concerning online mental health support for young people is still emerging,^36^ although their use of social media generates an anxious public discourse around risks posed by the spread of harmful ideas and connections.^37^ The concepts of suicide contagion and collusion illustrate these concerns. ‘Suicide contagion’ is understood theoretically as analogous to contagion in infectious disease, such that suicidality can be passed between individuals.^38^ For example, following a series of high profile youth suicides in the UK in 2007–08, and despite a lack of clarity about any role of social media in this case, the press speculated that contagion and collusion via social media had contributed to the tragedies.^39^ Similarly in relation to eating disorders, ‘collusion’ has been conceptualized as a mechanism by which harmful ideas are spread, as has been observed in pro‐eating disorder websites,^12^ so digital technologies may compromise the well‐being of some young people.

Despite the risks, some voices argue in favour of trusting young people to find ways to look after themselves.^25^, ^40^, ^41^ Young people are not necessarily passive consumers of Internet material and social media and can look critically at the content.^42^ Evidence from evaluations of structured, web‐based interventions has highlighted that digital health resources can be acceptable and accessible, offering significant health benefits for young people, including better clinical outcomes,^43^ enhanced health literacy^44^ and improved access to hard to reach groups.^45^

The literature shows that young people, and individuals with concerns about eating or eating disorders, appear to encounter specific helpseeking difficulties; but also that online support has potential to address some of the barriers faced by both groups.^34^ Therefore, online support may be especially useful to young people who have concerns about eating or eating disorders. So far, research in this field has focused on the evaluation of structured interventions^46^, ^47^, ^48^ or the potential dangers from unstructured discussion online (e.g., Ref.^16^) with little attention given to how young people use unstructured online resources to support their mental health. Additionally, little is known about how forum moderation might influence online discussion.^49^ In view of the importance of the topic and the scant evidence base, there is a pressing need for research in this area. No previous studies have explored in depth how young people use a moderated online discussion forum for support with eating disorders. Our study aimed to address these gaps in knowledge.

## Study aim and context

Our study aimed to explore how young people use an online discussion forum for support with eating disorders. We identified an eating disorders charity that provided a moderated online discussion forum, whose stated purpose was to facilitate support between young people with concerns about eating and eating disorders, who were seeking support and advice towards recovery. We focused on the messages posted on the forum (rather than the individuals posting them) and thus observed active participation through published messages; but lurking, that is, passive use of the forum, was not visible.^50^ Although the forum was accessible to the public, we obtained the charity's permission to study it, and for the duration of the data collection, we published an explanation about our research on the website. Box 1 summarizes key features of the online forum.

Box 1 **Key features of the online discussion forum**

**Description of eating disorders online discussion forum for young people**

- The forum is located within the website of a pro‐recovery eating disorders charity.
- The stated aim of the forum is to facilitate peer support between young people who have concerns about eating and are looking towards recovery.
- The forum supports asynchronous discussion in conversational threads.
- The forum is available 24 hours a day.
- The forum is publically accessible for reading and posting messages, via registration.
- The forum is moderated by charity employees.
- Content blocked if not pro‐recovery (e.g. collusion; triggers such as weight, or names of foods).
- Guidelines for using the forum clearly stated on the website:
  o Messages should be respectful and supportive.
  o Messages should avoid unhelpful language such as details about food, calories, symptoms, exercise or weight.
  o It is not permitted to reveal real names of people or places or to include any information that could lead to individuals being identified with an email.
- People writing to the forum are known by a username only.
- Forum users are unable to contact each other privately.
- A moderator who has concerns about a message can attempt to contact the individual by email, but has no other means of contact.

The purpose and process of moderation was stated clearly on the forum through a comprehensive set of guidelines for users accompanied by a carefully articulated rationale. Moderation was intended to keep users of the forum safe, and posts were edited if the moderators believed they contravened these guidelines. Users were asked to avoid naming people and places, including professionals and treatment centres; names of medication; medical advice; references to height and weight; types and names of food and drink; criticism of other forum users; discussion that encouraged users to compete, be dependent on each other, resist treatment or lose weight; graphic descriptions of behaviour (such as self‐harm) that could be distressing; references to media such as books, films and magazines that the moderators believe to be inappropriate for the forum; and copyrighted material.

Due to our study methods we did not know whether all individuals writing to the forum noticed this information; however, we did find brief references to moderation within the messages we scrutinized, which indicates that some were aware of it.

## Methods

### Study design

The study design was based on an online ethnographical, or netnographical, approach previously used by Kirk and Milnes.^51^ Netnography applies ethnographic approaches to online behaviour, drawing on publicly accessible online social interactions in the same way that an ethnographer observes human behaviour in the field.^52^

The research team included experienced researchers and practitioners with relevant knowledge about young people's mental and physical health and well‐being, eating disorders and analysis of online discussion forums, which informed our judgements throughout the study.

### Data collection

With the charity's permission, we collected all messages posted during a 4‐month period (August–November 2012). We downloaded the messages into Microsoft™ Word and imported them into the qualitative analysis software programme, NVIVO 10.^53^

### Sample

Posts appeared on the forum organized into broad topic areas. We discarded topics that were primarily concerned with the mechanisms of using the forum. This left seven topics, containing 119 unique usernames, 97 threads (i.e. subtopics) and 420 messages. However, the topic labels (Table 1) did not always reflect the content of the messages as users seemed to pick a specific topic as a ‘home’ for all the messages they posted, regardless of their focus at the time.

**Table 1:** Online discussion forum detail

Category	Posts (N)	Usernames (N)
How to use the message boards	66	22
Caring about someone	11	5
Eating disorders	156	59
Poetry	3	3
Introductions	83	23
Recovery	92	34
Inspiration	9	7
Total	420	153
Total unique usernames		119

Most user names occurred 1–4 times. The highest number of occurrences of one username was 64.

We did not have access to any detail beyond what was written in the messages that appeared on the board; however, there appeared to be a wide range of forum users including people just looking for advice, or with concerns about eating problems, as well as those dealing with severe eating disorders.

Some usernames appeared frequently and many only once. The greatest number of messages posted by a single user was 64, but the majority of users posted 1–4 times during the observational period. Table 1 contains further detail.

### Thematic analysis

The aim of the analysis was to understand the ways in which young people use an online discussion forum for support with eating disorders. We used a thematic analysis method suitable for qualitative data that enables rich and detailed interpretation.^54^ We were mindful that we only had access to constructed identities of the individuals who were commenting, and there was also the unknown impact of forum moderation; both factors are common in netnographic studies.^51^

First, Elvey and Kendal read and familiarized themselves with the data and generated initial codes reflecting the content. Next, they identified themes relevant to our aim of understanding the support mechanisms of the forum. The themes were reviewed and refined through discussion with the research team. Using this method, we identified five final themes that reflected how the forum seemed to be used (see Table 2).

**Table 2:** Final themes

Final themes
Taking on the role of mentor
The online forum as a safe space
Friendship within the online forum
Flexible help
Peer support for recovery and relapse prevention

### Ethical considerations

Online communities for young people are recognized as a valid focus for research,^55^ and the forum was in the public domain. However, there is also a debate on the ethical issues around using publicly accessible online discussion for research.^56^, ^57^ The identities of the forum users were unknown so we were not in a position to obtain individual consent from them. Instead, the charity gave us proxy consent to access and use the posts. To enhance transparency,^58^ we advertised and explained our research on the charity's website and Twitter feed before commencing the study. We protected the privacy of the forum users by removing terms and phrases that could identify them, including the name of the charity. We obtained ethical approval for this study in March 2012 from the UK NHS National Research Ethics Service and the University of Manchester Research Ethics Committee.

## Results

The charity's stated purpose for the online discussion forum was to facilitate peer support between young people with eating disorders, and our five themes each represented a dimension of peer support which individuals accessed through the forum: Taking on the role of mentor; The online forum as a safe space; Friendship within the online forum; Flexible help; and Peer support for recovery and relapse prevention (See Table 2).

In our presentation of results, quotations are reproduced verbatim to reflect the realism of Internet use, and usernames have been replaced with codes.

### Taking on the role of mentor

Forum users offered mentorship in the form of advice and encouragement, seemingly drawing on lived experiences of eating disorders. Some individuals appeared to value being able to mentor others, which reflects an established view that taking on responsibilities and helping others supports emotional well‐being.^40^

I'm only 10 so I'm one of the younger ones but if you need someone to talk to I'd be happy to hope your ok

(E1UN)

There were lengthy, reassuring messages to those who sounded lost and frightened. Suggestions included cognitive reframing, using diaries, crisis planning, working with goals, distraction and behavioural methods. Such advice appeared to be welcomed within the forum alongside tips for relaxation techniques and using creative arts for self‐expression.

Basically, if you can work on behaviour, emotions and thoughts will automatically start to improve

(E6UF)

Thank you for the advise and speaking of goals I made a goal to tell one of my friends what has happened, which is a big thing as I have'nt actually told any of my friends yet. But I finally did it

(E1UB)

…it will really be worth the hard work once you have mastered the art of relaxation (E1AL)

Although we had no information about people using the forum beyond what they chose to write, suggestions were often made with great confidence, so could have been drawn from personal experience of therapeutic interventions. Much of the advice was consistent with general self‐help principles in mental health,^59^ although posts did not reveal whether the writers had been taught these strategies or simply worked them out.

### The online forum as a safe space

The forum seemed to be a trusted environment for individuals to talk about isolation, fear, shame or despair in an atmosphere of mutual support and acceptance. Although this appears to be common to online discussion groups,^51^, ^60^, ^61^ it has particular relevance here, because the intense internal conflicts that characterize disordered eating seem difficult to articulate. The combination of anonymity and an expectation of acceptance may have mitigated barriers to connecting with other people.

I suffer from bulimia, and now after I purge, I have alot of discomfort…I dont feel I can tell my parents even though I really want my mum to help me. I feel so lonely and disgusting… I am too scared to go to a doctors I have no‐one to turn to and I have no idea what to do

(E4LO)

The moderation seemed to balance a light touch (e.g. the fact that messages were not organized into topics by moderators),^62^ with delays in publishing messages because of the need to moderate them first. In the brief exchange below, there is a reference to the impact of the forum's asynchronicity:

hey sorry about the late reply, i've only just been notified that people have written back to me

(E6IN)

don't worry i really don't mind, these messages have to checked and moderated and that normally takes time

(E6LI).

We also found fleeting references to the removal of trigger words, for example:

I have been known to snap at people, get aggressive and switch moods rapidly as well as suffer with the ED and other mental health conditions (Won't mention because of triggers) (E3LA)

Therefore, it seemed likely that there was an element of censorship or self‐censorship shaping what appeared on the forum. These characteristics suggest that its function as a safe space might depend on both moderation processes and forum culture, expressed through peer activity.

Forum users at the point of realising they had an eating disorder could use online discussion to get used to the idea and practise talking about it. Lack of control over confidentiality deters many young people from asking for help.^63^ The forum operated through anonymity, thus ensuring personal information could not be shared. In this respect, the forum offered support that would be difficult to find in face to face encounters.

### Friendship within the online forum

The sense of friendship within the forum was strong. Forum users wrote affectionately, for example

Hope your ok and having a good week xxxx

(E1UN)

I've not been on here in ages and was wondering if any of the old members were still using [the forum]? I would love to know if anyone has heard from [username]?! Hope everyone is doing okay

(E4AN)

Forum friendships offered emotional support and companionship. Messages were humorous, expressed excitement or described ordinary events without reference to problems with eating. Some commiserated about the difficulties of navigating family relationships. Celebrations that involved communal eating or being scrutinized by relatives could be anticipated with immense anxiety, and individuals seemed to trust the forum community to be empathic and constructive. The quotations below are a sample from an exchange between three individuals.

Hello guys… I'm fourteen years old and have suffered with anorexia since i was eleven…. i need some reassurance ‐ is this it? is this as good as it gets? is recovery possible… i mean FULL recovery… xxxxxxxx

(E7EC)

I know what you feel. I used to feel it too. I would say yes you can be fully recovered from an eating disorder, however it will be a very hard and long process I guess. I'm in the stage of recovering too

(E7WC)

…thank you for that wonderful advice! so what stage of recovery are you at?…

(E7EC)

…I believe full recovery is possible i am battling anorexia right now, but my mum suffered it and now she is a healthy happy person…

(E7AD)

…! thats wonderful that your mum is recovered! that must reassure you a litte! How old are you? How long have you been ill for? Xxx

(E7EC)

Hi! … i'm 11. I have been ill for about 6 months now. i have gone to therapy and stuff like that e.g. a dietitian. It's really nice and reassuring to know that you have been in a similar position to me … i hope you are doing really well!!

(E7AD)

hi (E7AD) I'm doing ok, I also have OCD which is really tricky?ive been in full time [school] now for the past eight weeks which has been hard and tiring but im proud!! … hope you're ok, keep fighting!! xxx

(E7EC)

### Flexible help

The online forum environment had the flexibility to respond to the needs of people at different points on a helpseeking journey. Although asynchronous, the forum was open all the time, unlike helplines or support groups whose availability is sometimes restricted. Again and again, individuals would be urged to find someone to tell and helped to think about ways they could create a situation for disclosure. If one forum user wrote that they had tried telling, and things had gone badly, another would encourage them to try again, with a different person or in a different way.

Perhaps if it seems to complicated to explain, you could give [your mum] a letter, ask her to read it, and speak to her after? This can give both of you time to process. Let me know how it goes!

(E3UF)

Denial is a part of it, and you just need to be brave and tell yourself that going to a doctor and admitting things are difficult is the way you can be happier

(E4AN)

Although people using the forum might also be accessing formal mental health services, the flow of helpseeking was in both directions, in that some users said they had been encouraged to access it by a health‐care practitioner. Indeed, the forum appeared to have credibility within formal services as an actual source of support, and not merely as a signpost to other services.

I was diagnosed with anorexia nervosa and now I see a phsyciatric nurse and have to be weighed each week. I just came onto this website as my psych reccomended it and it helps to see that your not the only one struggling

(E6EB)

Hey guys, just joined I'm 15 and i think i may have anorexia. I have not been diagnosed and no one else knows I was adviced to come here by Childline [UK helpline for children and YP] and i am too scared to tell anyone how i feel. My parents would never understand, nor my friends, what can i do?? I want to stop

(E4MU)

Messages described how it felt to find the courage to seek help from close friends and family, or access formal services. Forum users encouraged each other to keep going in the face of initially unhelpful professional input, while acknowledging that sometimes health‐care professionals signposted them to the forum.

I've just joined this site and came across your post (sorry i hope its not too late to help). The first time i went to my Doctor after going through alot of suffering before admitting i had a problem. They gave me a HEALTHY FOOD CHART and sent me away. i felt so stupid and hopeless…It wasnt until they started treating me for depression that they realised the real route of my depression was actually my ED, and i eventually got referred to the help i needed. Please do not give up.

Messages also described finding effective help outside mental health services.

Yeah, it was a Christian camp which my youth group go to every year but I'd never been before. It was amazing and I had loads of prayer for my condition and I feel as though I have been healed

(E6OZ)

One described how they had accessed college support after being encouraged to do so on the forum.

I really can't express how happy I am that I spoke to my college about it

(E4AN)

### Peer support for recovery and relapse prevention

For people in recovery, the forum appeared to help by facilitating mutual support and encouragement. It was common for forum users to refer to an inner voice that told them not to eat and made them feel guilty if they did. Part of recovery was anticipating when the voice would re‐emerge and bring feelings of obsession with eating/not eating, guilt and shame.

it feels like a trap! you think your fine and then it just goes ‘boom! i'm back!’

(E6LI)

its like a feeling of controllment in my head, if you understand what I mean? always saying to itself all the negative thoughts about my body, food, exercise and what I can eat when etc.

(E6IN)

In some aspects, the forum appeared to present eating disorders as a chronic illness, that is with periods of better/worse health. It included individual stories of severe illness from people who were struggling against relapse and seeking help with coping. This included very young forum users.

i'm only 10 but i'm trying to get better from my eating disorder too … never give up hope everyone has a chance to recover

(E4UN)

I recently became ill with a bad kidney infection which left me in hospital and has started to bring out my anorexia tendencies…and my old Ed thoughts are creeping back.

(E4XS)

Hey i've been suffering with anorexia for nearly 3 years now. ive been in a secure unit twice. my anorexia last year turned into bulimia, and im still strugglying with it now … i restrict, then binge, then self‐harm… and im in this cycle constantly. my moods so low cause of how guilty i feel, the weights going up, and i cant control it… any advice? Xxxx

(E4EN)

The impact of longstanding fear of relapse was illustrated by forum users’ reported reservations about intimate relationships, indicating an awareness of long‐term social and emotional impact of eating disorders.

it sounds good that you have a relationship, i am always scared to enter one in case the boy doesn't understand the way i act like i do

(E6LI)

Some posts conveyed an impression that the writer was struggling with deteriorating health from their eating disorder. Posts suggested that individual health needs changed; for instance, community mental health services could be experienced as unhelpful at one point but helpful later on.

I recently went to the doctor and asked for help and after being referred on, i was told that I can't be helped! … now I'm scared that I am back to dealing with it on my own again. Is there anybody out there that can relate to this?

(E4LE)

i ama 14 year old girl i have had annorexia for about 2 years now unfortunatley after i came out of my inpatient unit i have find it quite hard. you may be thinking i have had a relapse and am now suddenly being threatend with in patient treatment again. its not quite like that. its just that there every now and again i get these little nudges backwards and slowly i am going back down hill

(E3XP)

## Discussion

The contribution of this study is to generate knowledge about how young people use an online discussion forum for support with eating disorders. As such, our study met its aims. We found that the forum had some key features that seemed to address help‐seeking barriers: enabling individuals to take on the role of mentor; being a safe space for discussion, facilitating friendship, offering help flexible enough to fit with individual need, and providing a platform for peer support towards recovery. Laing *et al*.'s^33^ model for helpseeking (defining, deciding, selecting) provides a useful framework for discussing the findings.

Mentorship, help‐seeking and peer support are familiar themes in the context of self‐care support, but have not previously been explored in relation to online support for eating disorders. We have argued that the egosyntonic nature of many eating disorders, and the developmental and contextual circumstances of young people, present specific barriers for young people with concerns around eating, who are seeking support. One of the messages quoted here described relapse as something in their head that appeared ‘without warning’. According to Tierney and Fox,^64^ there may be potential in therapeutic approaches that acknowledge and discuss the inner voice, following advances made in treatment of psychosis. Being able to talk about the anorexic voice on the forum could help dissipate some of its power.

### Defining the problem

The online discussion forum provided a safe space for individuals to ventilate their feelings without having to deal with other people's reactions. It allowed anonymity, which for some people seemed to be instrumental in enabling them to acknowledge and articulate a frightening problem. They could access friendship, peer support and mentorship to help them reflect on their experiences and other people's, ultimately being able to define a problem that they could then go on to address.

Self‐disclosure is a recurring theme in the literature around online support for eating disorders.^65^ linked lack of self‐disclosure to denial and treatment resistance, an insight that can help address the egosyntonic picture that makes engagement in treatment so challenging.^18^, ^66^ Sharing experiences was also identified as important in a study of eating disorder discussion boards on Yahoo!^67^ and in pro‐Anamia blogs.^68^ Tong *et al*.^68^ found that reciprocal self‐disclosure was a common form of social support, and argued that being able to ventilate feelings during a period of stress had therapeutic merit. Despite these positive perspectives, the literature also highlights a need for strategies to evaluate the quality of online support for eating disorders, to mitigate the risks posed from collusion and contagion.^16^

Peer support may be attractive because there is an expectation of mutual understanding and a lesser power differential between helper and help‐seeker.^69^ However, young people may not perceive their peers as trustworthy.^70^ The literature consistently shows that young people worry that anyone they approached for emotional support could compromise them by sharing their personal and sensitive information with others.^24^, ^30^, ^31^, ^32^, ^70^, ^71^, ^72^ The peer support offered on the online forum may be attractive specifically because of its certain anonymity, echoing the findings of other studies of online discussion.^43^, ^44^, ^45^, ^73^

### Deciding to seek help

Forum users seemed to engage with the friendship, peer support and mentorship communicated through the messages, to learn about different kinds of help or how people have coped when helpseeking has gone wrong. They could choose their level of engagement and be non‐committal about accessing support, which for some might be the start of a recovery journey.

Young people's agency tends to arouse conflicting emotions, reflecting perhaps their social status as both vulnerable and independent. It can be tempting to prioritize safety over risk‐ the implication being that young people should be protected from engaging with strangers. Yet arguably, it is the young person who should decide what ‘safety’ means. For instance, young people often value their place within a peer group,^20^ so they could experience social isolation as far more dangerous to well‐being than belonging to a community, even a virtual one.

Therefore, our findings support the paradox that, while a young person can engage in risky behaviour,^25^ their agency is, in itself, an important facilitator of help‐seeking for emotional support and can promote their emotional well‐being.^34^, ^74^, ^75^, ^76^ Little is known about the specific support preferences of young people with eating disorders, but considering the developmental^20^ and egosyntonic^18^ factors, the availability, accessibility, privacy and inclusiveness of the forum may have made it a flexible and attractive form of support. Given the associated risks^16^, ^36^ it may seem counter‐intuitive to direct young people to an online discussion forum; yet it may ultimately enhance subjective well‐being, even though the conversations are effectively with a stranger whom one has met on the internet, and a sure way to inspire moral panic in the media.

### Selecting a source of support

Our findings support the literature suggests that developmentally appropriate emotional support for young people enhances choice, privacy and control over the consequences of help‐seeking.^58^, ^74^, ^77^, ^78^ Although it did not offer an immediate response, its accessibility at any time may have been important to individuals who valued being able to choose when to use it.

Amongst the advice about encouraging people to seek advice from health professionals, substantial discussion was around co‐production of care, which promotes agency and conceptualizes the user as an expert. The data illustrated how individuals could engage with formal services, peers and their own internal processing on their own terms, to work towards outcomes that they valued.

As all the messages posted on the forum had been subject to moderation, it is unsurprising that none were unfriendly in tone or not directed towards recovery. Giles's analysis of web discourses around eating disorders found tensions within the online community^17^ but our data, subject to forum moderation, did not show this. Although there appeared to be a light touch approach,^62^ moderation could however also be perceived as undermining forum users’ ownership of their contributions and discussions. The tension between these two positions has been acknowledged in the literature.^41^

The literature highlights synergy between positive help‐seeking in young people and having the freedom to make health choices^25^; according to our data, young people were being signposted there by health professionals operating within formal services‐ perhaps because they trusted it. Forms of support that have been demonstrated to help young people towards recovery from emotional difficulties include friendship,^60^, ^79^, ^80^ with advice,^81^ peer support^69^, ^82^ and space to express difficult thoughts and feelings.^83^ These characteristics have also been highlighted as valued by young people with other conditions, such as asthma.^84^ Such support may in turn encourage personal attributes that have been linked with emotional well‐being, including connectedness, effective coping strategies, health literacy,^75^ helping others^40^; positive emotions, hope and social attachments.^74^ Therefore, we argue that this moderated online discussion forum can be conceptualized as a positive agent for young people's well‐being, promoting constructive decision‐making and self‐care.

## Strengths and limitations

The contribution of this study is to generate knowledge about how young people use an online discussion forum for support with eating disorders. It highlights the potential of digital media to support young people's emotional health. The issue is poorly understood. We have argued that a young person's decision to use this form of social media can be construed as a positive action that promotes their self‐care and encourages helpseeking. As such, the findings counter negative media perceptions of online groups.

Our findings may reflect the views of individuals who have already overcome potential barriers to helpseeking. It is a limitation of our study and web‐based health support in general.

It was beyond the scope of this study to evaluate the impact of moderation and we do not know how the fact that there was moderation influenced what people posted or what appeared on the forum. Our focus was the messages and we did not set out to interview young people on this issue. Apart from rare, brief references to the fact that the forum was moderated, we did not find discussion about this in our dataset.

There is limited research into the moderation of online discussions, although there are indications that timely responses and forum moderation influence engagement.^85^, ^86^ In common with online research, we took our data at face value. The dataset was from a 4‐month period and any seasonal trends in the posts would not be identified from this study. Another issue to be explored through further study is the level of skill (emotional, literary and practical) and resources (opportunities to access the internet) that a board user is demonstrating by creating a post. Clearly these topics warrant further investigation. There are substantial implications for health and health care, which illustrate a pressing need for the development of research in this field.

## Conclusion

Our study illustrates how young people use an online discussion forum to help themselves to manage or recover from eating disorders – with, without or despite clinical interventions. We suggest that it facilitates support characterized by less control from others, which allows more control over process by a young person and hence fewer barriers to helpseeking and more engagement in personal recovery.

Our findings may help reassure carers, practitioners and users that online discussion forums can be safe and therapeutic environments for young people with eating disorders. Forum moderation may be a key influence on the therapeutic potential of this form of media. Further research is needed to improve understanding of forum moderation and other factors that influence the safety of young people accessing emotional support online.

## References

[1] World Health Organization (WHO) . International Statistical Classification of Diseases and Related Health Problems 10th Revision (ICD‐10) Version for 2010: WHO, 2015, [updated 2015]. Available at: http://apps.who.int/classifications/icd10/browse/2015/en, accessed 17 February 2015.

[2] Wittchen HU , Jacobi F , Rehm J *et al* The size and burden of mental disorders and other disorders of the brain in Europe 2010. ECNP/EBC REPORT European Neuropsychopharmacology, 2011; 21: 655–679. 10.1016/j.euroneuro.2011.07.018 21896369

[3] Hudson JI , Hiripi E , Pope HG , Kessler RC . The prevalence and correlates of eating disorders in the national comorbidity survey replication. Biological Psychiatry, 2007; 61: 348–358. 16815322 10.1016/j.biopsych.2006.03.040 PMC1892232

[4] Preti A , Girolamo Gd , Vilagut G *et al* The epidemiology of eating disorders in six European countries: results of the ESEMeD‐WMH project. Journal of Psychiatric Research, 2009; 43: 1125–1132. 19427647 10.1016/j.jpsychires.2009.04.003

[5] World Health Organization (WHO) . Adolescent Health 2015. Available at: http://www.who.int/topics/adolescent_health/en/, accessed 28 February 2015.

[6] Micali N , Hagberg KW , Petersen I , Treasure JL . The incidence of eating disorders in the UK in 2000–2009: findings from the General Practice Research Database. BMJ Open, 2013; 3:5 e002646 doi:10.1136/bmjopen‐2013‐002646. 10.1136/bmjopen-2013-002646 PMC3657659 23793681

[7] Berkman ND , Lohr KN , Bulik CM . Outcomes of eating disorders: a systematic review of the literature. International Journal of Eating Disorders, 2007; 40: 293–309. 17370291 10.1002/eat.20369

[8] Fairburn C , Christopher GF , Paul JH . Eating disorders. The Lancet (British Edition), 2003; 361: 407. 10.1016/S0140-6736(03)12378-1 12573387

[9] Hillege S , Beale B , McMaster R . Impact of eating disorders on family life: individual parents’ stories. Journal of Clinical Nursing, 2006; 15: 1016–1022. 16879546 10.1111/j.1365-2702.2006.01367.x

[10] Polivy J , Herman PC . Mental health and eating behaviours: a bi‐directional relation. Canadian Journal of Public Health/Revue Canadienne de Sante'e Publique. 2005;96 (Supplement 3: Understanding the Forces That Influence Our Eating Habits: What We Know and Need to Know (JULY/AUGUST 2005)):3. 10.1007/BF03405201 PMC6975888 16042164

[11] beat . beat: beating eating disorders UK: beat, 2010 Available at: http://www.b-eat.co.uk/, accessed 22 May 2014.

[12] Dias K . The Ana sanctuary: women's pro‐anorexia narratives in cyberspace. Journal of International Women's Studies, 2003; 4: 31–45.

[13] Burke E . Reflections on the waif. Australian Feminist Studies, 2012; 27: 37–54.

[14] Casilli AA , Tubaro P , Araya P . Ten years of Ana: lessons from a transdisciplinary body of literature on online pro‐eating disorder websites. Social Science Information, 2012; 51: 120–139.

[15] Jett SK . Impact of exposure to pro‐eating disorder websites on body dissatisfaction and eating behavior in college women. Dissertation Abstracts International: Section B: The Sciences and Engineering. 2008;69(2‐B):1328.

[16] Rouleau CR , von Ranson KM . Potential risks of pro‐eating disorder websites. Clinical Psychology Review, 2011; 31: 525–531. 21272967 10.1016/j.cpr.2010.12.005

[17] Giles D . Constructing identities in cyberspace: the case of eating disorders. British Journal of Social Psychology, 2006; 45: 463–477. 16984715 10.1348/014466605X53596

[18] Westwood LM , Kendal SE . Adolescent client views towards the treatment of anorexia nervosa: a review of the literature. Journal of Psychiatric and Mental Health Nursing, 2012; 19: 500–508. 22070426 10.1111/j.1365-2850.2011.01819.x

[19] Blades R , Renton Z , La Valle I , Clements K , Gibb J , Lea J . We Would Like to Make a Change. London: National Children's Bureau, 2013.

[20] Coleman JC . The Nature of Adolescence. London: Routledge, 2011.

[21] Higbed L , Fox J . Illness perceptions in anorexia nervosa: a qualitative investigation. British Journal of Clinical Psychology, 2010; 49(Pt 3): 307–325. 19580703 10.1348/014466509X454598

[22] Rieger E , Touyz S , Swain T , Beumont P . Cross‐cultural research in anorexia nervosa: assumptions regarding the role of body weight. International Journal of Eating Disorders, 2001; 29: 205–215. 11429983 10.1002/1098-108x(200103)29:2<205::aid-eat1010>3.0.co;2-1

[23] Boyd C , Hayes L , Nurse S *et al* Preferences and intention of rural adolescents toward seeking help for mental health problems. Rural and Remote Health [Internet]. 2011; 11: [e1582 p.] Available at: http://www.pubfacts.com/detail/21319934/Preferences-and-intention-of-rural-adolescents-toward-seeking-help-for-mental-health-problems. 21319934

[24] Gulliver A , Griffiths KM , Christensen H . Perceived barriers and facilitators to mental health help‐seeking in young people: a systematic review. BMC Psychiatry, 2010; 10: 113. 21192795 10.1186/1471-244X-10-113 PMC3022639

[25] Wilson CJ , Rickwood DJ , Bushnell JA , Caputi P , Thomas SJ . The effects of need for autonomy and preference for seeking help from informal sources on emerging adults’ intentions to access mental health services for common mental disorders and suicidal thoughts. Advances in Mental Health, 2011; 10: 29–38.

[26] YoungMinds . YoungMinds written submission to the House of Commons Health Select Committee on Child and Adolescent Mental Health Services. Young Minds 2014.

[27] Van Der Riet M , Knoetze J . Help seeking patterns in urban and rural youth in two South African provinces: a socio‐contextual View. School Psychology International, 2004; 25: 223–240.

[28] Sears HA . Adolescents in rural communities seeking help: who reports problems and who sees professionals? Journal of Child Psychology & Psychiatry, 2004; 45: 396–404. 14982252 10.1111/j.1469-7610.2004.00230.x

[29] Sourander A , Helstelä L , Ristkari T , Ikäheimo K , Helenius H , Piha J . Child and adolescent mental health service use in Finland. Social Psychiatry and Psychiatric Epidemiology, 2001; 36: 294–298. 11583459 10.1007/s001270170047

[30] Mizuno H , Ishikuma T , Tamura S . Help‐seeking preferences of junior high school students in Japan. Japanese Journal of Counseling Science, 2006; 39: 17–27.

[31] Kendal S , Keeley P , Callery P . Student help seeking from pastoral care in UK high schools: a qualitative study. Child and Adolescent Mental Health, 2014; 19: 178–184. 10.1111/camh.12029 32878370

[32] Freedenthal S , Stiffman AR . “They might think I was crazy”: young American Indians’ reasons for not seeking help when suicidal. Journal of Adolescent Research, 2007; 22: 58–77.

[33] Liang B , Goodman L , Tummala‐Narra P , Weintraub S . A theoretical framework for understanding help‐seeking processes among survivors of intimate partner violence. American Journal of Community Psychology, 2005; 36: 71–84. 16134045 10.1007/s10464-005-6233-6

[34] Pryjmachuk S , Elvey R , Kirk S , Kendal S , Bower P , Catchpole R . Developing a model of mental health self‐care support for children and young people through an integrated evaluation of available types of provision involving systematic review, meta‐analysis and case study. Health Services & Delivery Research, 2014; 2. 25642499

[35] Berriman L , Thomson R . Spectacles of intimacy? Mapping the moral landscape of teenage social media. Journal of Youth Studies, 2014; 18: 583–597.

[36] Kauer SD , Mangan C , Sanci L . Do online mental health services improve help‐seeking for young people? A systematic review Journal of Medical Internet Research, 2014; 16: e66. 24594922 10.2196/jmir.3103 PMC3961801

[37] Eichenberg C . Internet message boards for suicidal people: a typology of users. Cyberpsychology and Behavior, 2008; 11: 107–113. 18275323 10.1089/cpb.2007.9924

[38] Gould M , Jamieson P , Romer D . Media contagion and suicide among the young. American Behavioral Scientist, 2003; 46: 1269–1284.

[39] Jones P , Gunnell D , Platt S *et al* Identifying probable suicide clusters in Wales using national mortality data. PLoS One, 2013; 8: 8. e71713. doi: 10.1371/journal.pone.0071713. eCollection 2013. 10.1371/journal.pone.0071713 PMC3756004 24015189

[40] Hart A , Aumann K , Heaver B . Boingboing resilience research and practice 2010 [updated 2015]. Available at: www.boingboing.org.uk, accessed 18 August 2015.

[41] Staksrud E , Livingstone S . Children and online risk: powerless victims or resourceful participants? Information, Communication & Society, 2009; 12: 364–387.

[42] Henderson EM , Eccleston C . An online adolescent message board discussion about the internet: use for pain. Journal of Child Health Care: For Professionals Working With Children in the Hospital and Community, 2013; 19: 412–418. doi:10.1177/1367493513509420. 24270989 10.1177/1367493513509420

[43] Stinson J , Wilson R , Gill N , Yamada J , Holt J . A systematic review of internet‐based self‐management interventions for youth with health conditions. Journal of Pediatric Psychology, 2009; 34: 495–510. 19029142 10.1093/jpepsy/jsn115

[44] Guse K , Levine D , Martins S *et al* Interventions using new digital media to improve adolescent sexual health: a systematic review. Journal of Adolescent Health, 2012; 51: 535–543. 23174462 10.1016/j.jadohealth.2012.03.014

[45] Aardoom JJ , Dingemans AE , Spinhoven P , Van Furth EF . Treating eating disorders over the internet: a systematic review and future research directions. International Journal of Eating Disorders, 2013; 46: 539–552. 23674367 10.1002/eat.22135

[46] Boydell KM , Hodgins M , Pignatiello A , Teshima J , Edwards H , Willis D . Using technology to deliver mental health services to children and youth: a scoping review. Journal of the Canadian Academy of Child and Adolescent Psychiatry, 2014; 23: 87–99. 24872824 PMC4032077

[47] Clarke AM , Kuosmanen T , Barry MM . A systematic review of online youth mental health promotion and prevention interventions. Journal of Youth and Adolescence, 2015; 44: 90–113. 25115460 10.1007/s10964-014-0165-0

[48] Hieftje K , Edelman EJ , Camenga DR , Fiellin LE . Electronic media‐based health interventions promoting behavior change in youth: a systematic review. JAMA Pediatrics, 2013; 167: 574–580. 23568703 10.1001/jamapediatrics.2013.1095 PMC3733329

[49] Matzat U , Rooks G . Styles of moderation in online health and support communities: an experimental comparison of their acceptance and effectiveness. Computers in Human Behavior, 2014; 36: 65–75.

[50] Schneider A , von Krogh G , Jäger P . “What's coming next?” Epistemic curiosity and lurking behavior in online communities. Computers in Human Behavior, 2013; 29: 293–303.

[51] Kirk S , Milnes L . An exploration of how young people and parents use online support in the context of living with cystic fibrosis. Health Expectations, 2015; Feb 17. doi: 10.1111/hex.12352. [Epub ahead of print] 10.1111/hex.12352 PMC5055278 25691209

[52] Salzmann‐Erikson M , Eriksson H . LiLEDDA: a six step forum‐based netnographic research method for nursing science. Aporia, 2012; 4: 7–18.

[53] QSR International . NVIVO 10. QSR International, Warrington, UK.

[54] Braun V , Clarke V . Using thematic analysis in psychology. Qualitative Research in Psychology, 2006; 3: 77–101.

[55] Montgomery K , Gottlieb‐Robles B . Youth as e‐citizens: the internet's contribution to civic engagement In: Buckingham D, Willett R (ed.) Digital Generations: Children, Young People, and New Media. London: Lawrence Erlbaum Associates Publishers, 2006: 131–148.

[56] Bond C , Ahmed O , Hind M , Thomas B . The conceptual and practical ethical dilemmas of using health discussion board posts as research data. Journal of Medical Internet Research, 2013; 15: e112. 23748147 10.2196/jmir.2435 PMC3713935

[57] Kozinets R . Netnography: Doing Ethnographic Research Online. London: Sage, 2010.

[58] Driver S . Virtually queer youth communities of girls and birls: dialogical spaces of identity work and desiring exchanges In: Buckingham D, Willett R (eds) Digital Generations: Children, Young People and New Media. Mahwah, NJ, USA: Lawrence Erlbaum Associates Publishers, 2006: 229–245.

[59] Lovell K , Bee P , Richards D , Kendal S . Self‐help for common mental health problems: evaluating service provision in an urban primary care setting. Primary Health Care Research and Development, 2006; 7: 211–220.

[60] Sherman LE , Greenfield PM . Forging friendship, soliciting support: a mixed‐method examination of message boards for pregnant teens and teen mothers. Computers in Human Behavior, 2013; 29: 75.

[61] Trondsen MV , Tjora A . Communal normalization in an online self‐help group for adolescents with a mentally ill parent. Qualitative Health Research, 2014; 24: 1407–1417. 25147219 10.1177/1049732314547708

[62] Comfort A . Moderating internal discussion forums, blogs and other social media Australia: Step Two, 2015 Available at: http://www.steptwo.com.au/papers/kmc_onlinemoderation/, accessed 20 August 2015.

[63] Rickwood D , Deane F , Wilson C . When and how do young people seek professional help for mental health problems? Medical Journal of Australia, 2007; 187(7 Suppl): S35–S39. 17908023 10.5694/j.1326-5377.2007.tb01334.x

[64] Tierney S , Fox JRE . Living with the ‘anorexic voice’: a thematic analysis. Psychology and Psychotherapy, 2010; 83: 243–254. 20109280 10.1348/147608309X480172

[65] Vandereycken W , Van Humbeeck I . Denial and concealment of eating disorders: a retrospective survey. European Eating Disorders Review: The Journal of the Eating Disorders Association, 2008; 16: 109–114. 18240122 10.1002/erv.857

[66] Abbate‐Daga G , Amianto F , Delsedime N , De‐Bacco C , Fassino S . Resistance to treatment in eating disorders: a critical challenge. BMC Psychiatry, 2013; 13: 294. 24229426 10.1186/1471-244X-13-282 PMC4225668

[67] Eichhorn KC . Soliciting and providing social support over the internet: an investigation of online eating disorder support groups. Journal of Computer‐Mediated Communication, 2008; 14: 67–78.

[68] Tong ST , Heinemann‐LaFave D , Jeon J , Kolodziej‐Smith R , Warshay N . The use of pro‐ana blogs for online social support. Eating Disorders, 2013; 21: 408–422. 24044597 10.1080/10640266.2013.827538

[69] Stanton‐Salazar RD , Spina SU . Adolescent peer networks as a context for social and emotional support. Youth & Society, 2005; 36: 379–417.

[70] Leach LS , Rickwood DJ . The impact of school bullying on adolescents’ psychosocial resources and help‐seeking intentions. Advances in School Mental Health Promotion, 2009; 2: 30–39.

[71] Cigularov K , Chen PY , Thurber BW , Stallones L . What prevents adolescents from seeking help after a suicide education program? Suicide & Life‐Threatening Behavior, 2008; 38: 74–86. 18355110 10.1521/suli.2008.38.1.74

[72] Wisdom JP , Clarke GN , Green CA . What teens want: barriers to seeking care for depression. Administration & Policy in Mental Health, 2006; 33: 133–145. 16489480 10.1007/s10488-006-0036-4 PMC3551284

[73] Aardoom J , Dingemans A , Boogaard L , Van Furth E . Internet and patient empowerment in individuals with symptoms of an eating disorder: a cross‐sectional investigation of a pro‐recovery focused e‐community. Eating Behaviors, 2014; 15: 350–356. 25064280 10.1016/j.eatbeh.2014.04.003

[74] Herrman H , Stewart D , Diaz‐Granados N , Berger E , Jackson B , Yuen T . What is resilience? Canadian Journal of Psychiatry, 2011; 56: 258–265. 21586191 10.1177/070674371105600504

[75] Rew L , Horner SD . Youth resilience framework for reducing health‐risk behaviors in adolescents. Journal of Pediatric Nursing, 2003; 18: 379–388. 15058534 10.1016/s0882-5963(03)00162-3

[76] Rutter M . Psychosocial resilience and protective mechanisms. American Journal of Orthopsychiatry, 1987; 57: 316–331. 3303954 10.1111/j.1939-0025.1987.tb03541.x

[77] Mustanski B , Lyons T , Garcia SC . Internet use and sexual health of young men who have sex with men: a mixed‐methods study. Archives of Sexual Behavior, 2011; 40: 289–300. 20182787 10.1007/s10508-009-9596-1 PMC2914836

[78] Sueki H , Eichenberg C . Suicide bulletin board systems comparison between Japan and Germany. Death Studies, 2012; 36: 565–580. 24563935 10.1080/07481187.2011.584012

[79] Erath SA , Flanagan KS , Bierman KL , Tu KM . Friendships moderate psychosocial maladjustment in socially anxious early adolescents. Journal of Applied Developmental Psychology, 2010; 31: 15–26.

[80] Philip K , Spratt J . Choosing your friends: young people negotiating supporting relationships. Advances in School Mental Health Promotion, 2010; 3: 42–51.

[81] Havas J , de Nooijer J , Crutzen R , Feron F . Adolescents’ views about an internet platform for adolescents with mental health problems. Health Education, 2011; 111: 164–176.

[82] Stjernswärd S , Ostman M . Illuminating user experience of a website for the relatives of persons with depression. International Journal of Social Psychiatry, 2011; 57: 375–386. 20233895 10.1177/0020764009358388

[83] Dolev‐Cohen M , Barak A . Adolescents’ use of Instant Messaging as a means of emotional relief. Computers in Human Behavior, 2013; 29: 58.

[84] Kirk S , Beatty S , Callery P , Milnes L , Pryjmachuk S . Perceptions of effective self‐care support for children and young people with long‐term conditions. Journal of Clinical Nursing, 2012; 21: 1974–1987. 22672459 10.1111/j.1365-2702.2011.04027.x

[85] Syred J , Naidoo C , Woodhall SC , Baraitser P . Would you tell everyone this? Facebook conversations as health promotion interventions Journal of Medical Internet Research, 2014; 16: e108. 24727742 10.2196/jmir.3231 PMC4042608

[86] Wise K , Hamman B , Thorson K . Moderation, response rate, and message interactivity: features of online communities and their effects on intent to participate. Journal of Computer‐Mediated Communication, 2006; 12: 24–41.

